# RADAR: a rigorously annotated database of A-to-I RNA editing

**DOI:** 10.1093/nar/gkt996

**Published:** 2013-10-25

**Authors:** Gokul Ramaswami, Jin Billy Li

**Affiliations:** Department of Genetics, Stanford University, Stanford, CA 94305, USA

## Abstract

We present RADAR—a rigorously annotated database of A-to-I RNA editing (available at http://RNAedit.com). The identification of A-to-I RNA editing sites has been dramatically accelerated in the past few years by high-throughput RNA sequencing studies. RADAR includes a comprehensive collection of A-to-I RNA editing sites identified in humans (*Homo sapiens*), mice (*Mus musculus*) and flies (*Drosophila melanogaster*), together with extensive manually curated annotations for each editing site. RADAR also includes an expandable listing of tissue-specific editing levels for each editing site, which will facilitate the assignment of biological functions to specific editing sites.

## INTRODUCTION

RNA editing is the post- or co-transcriptional modification of RNA nucleotides from their genome-encoded sequence. The most common type of editing in metazoans is the deamination of adenosine into inosine (A-to-I) catalyzed by the adenosine deaminase acting on RNA (ADAR) family of enzymes (1). ADAR enzymes bind double-stranded regions of RNA molecules and deaminate adenosine into inosine, which is subsequently recognized as guanosine by the cellular machinery. ADARs perform critical functions in the nervous system (2), and knockout of ADARs in mice causes lethality (1).

Historically, the identification of A-to-I editing sites has been dependent on the sequencing technologies available at the time. When DNA sequencing technologies were first being developed and automated, the identification of editing sites was slow and often occurred serendipitously. The development and growth of nucleotide databases facilitated the identification of additional editing sites. In recent years, the advent of high-throughout RNA sequencing (RNA-seq) has enabled transcriptome-wide identification of RNA editing sites and has greatly accelerated the discovery of A-to-I editing sites.

The major challenges in the field are to understand how RNA editing is regulated and to assign biological functions to specific editing sites. Currently, the widely used database of A-to-I editing sites is the database of RNA editing (DARNED) (http://darned.ucc.ie) (3). Although DARNED is a centralized repository for the location of A-to-I editing sites in the transcriptome, it contains few manually curated annotations and does not contain any information at all about the dynamic regulation of editing sites. RNA editing is tightly regulated in a spatiotemporal manner (4), and to elucidate the function of a particular editing site, it will be vital to analyze tissue-specific editing levels. We designed a rigorously annotated database of A-to-I RNA editing (RADAR) with this goal in mind. First and foremost, RADAR is an updated repository of A-to-I editing sites in humans, mice and flies. We included detailed manually curated annotations for each editing site as described later (see Database Features). In addition, for each editing site, we included a catalog of tissue-specific editing levels from published RNA-seq datasets. As further RNA-seq studies are published, the number of identified editing sites as well as the catalog of tissue-specific editing levels will be continuously updated to facilitate a deeper understanding of how RNA editing is dynamically regulated.

### Data collection

We collected a list of A-to-I editing sites in humans, mice and flies after performing a literature search. The first mammalian A-to-I editing sites were identified as amino acid recoding modifications in glutamate and serotonin receptors in the nervous system (5–7). As nucleotide sequences began to be deposited in expressed sequence tag (EST) databases, these resources were mined to identify additional A-to-I editing sites, focusing on editing events that changed amino acid sequences (8–12). EST database mining also demonstrated that A-to-I editing is quite prevalent in human Alu repeats (13,14). Additionally, a biochemical method to identify inosine in RNA molecules was developed by Sakurai *et al.* (15) and used to identify ∼5000 editing sites.

The vast majority of A-to-I editing sites have been identified in the past 2 years using high-throughput RNA-seq technologies. In humans, we first applied high-throughput sequencing to study A-to-I RNA editing by using a combination of targeted capture with padlock probes and high-throughput sequencing to identify several hundred editing sites (16). This success was followed by efforts to identify RNA editing sites in an unbiased transcriptome-wide manner by comparing sequence differences between matched RNA and DNA sequencing of a single individual. The first of these efforts (17) was controversial in that it claimed to provide evidence to support RNA editing of all 12 possible mismatch types, but further analyses (18–22) demonstrated that these non-canonical editing mismatches were false positives. Subsequent studies by us and others (23–26) developed meticulous computational pipelines to accurately identify A-to-I editing sites from matched RNA and DNA sequencing of human cell lines while minimizing technical artifacts from sequencing or read mapping errors. More recently, we developed a method to identify RNA editing sites using RNA-seq data alone by comparing transcriptome variants between different individuals (27). We used this method to identify A-to-I editing sites using RNA-seq data from human primary tissues whose genome sequencing data were not available (27). In total, at the time of first release, RADAR contains information describing 1 379 403 human A-to-I RNA editing sites.

In mice, Neeman *et al.* (28) identified clustered RNA editing sites from EST databases, and Danecek *et al.* (29) identified RNA editing sites using matched RNA and DNA sequencing data from brain tissues of 15 inbred mouse lines. In flies, Graveley *et al.* (30) identified RNA editing sites using RNA sequencing data from the modENCODE consortium, Rodriguez *et al.* (31) identified RNA editing sites using sequencing of nascent RNA transcripts and we (27) identified RNA editing sites using a comparative transcriptome method between three different *Drosophila* species. In total, at the time of first release, RADAR contains information describing 8108 mouse and 2698 fly A-to-I RNA editing sites.

### Database features

The genomic coordinates for all editing sites were first mapped onto the latest genome assemblies (human–hg19, mouse–mm9 and fly–dm3) using the liftOver tool from the University of California, Santa Cruz (UCSC) genome browser (32). For each editing site, we manually curated annotations, which consist of the genome assembly strand, associated gene, functional region within the gene (coding sequence, untranslated region, intron), associated repetitive element, conservation of editing to other species and the reference study in which the site was first identified.

**Figure 1.**
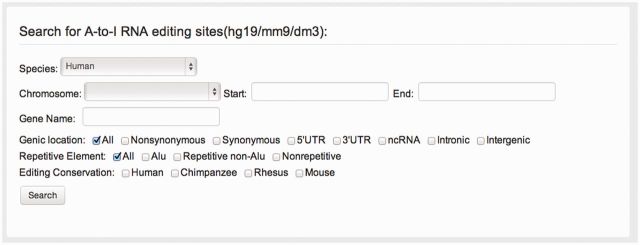
RADAR search page. Users can search for A-to-I editing sites in humans, mice or flies by any combination of the provided annotations: genomic location, gene, genic location, repetitive element overlap and editing conservation.

We designed a user-friendly web interface to query the database. The search page is displayed in Figure 1. Users must choose a species (human, mouse or fly) to search within. Users can filter their desired search using any combination of the listed annotations consisting of location in genome, gene, genic location (non-synonymous, synonymous, 5′-UTR, 3′-UTR, non-coding RNA, intronic, intergenic), repetitive element (Alu, repetitive non-Alu, nonrepetitive) and editing conservation (chimpanzee, rhesus and/or mouse for human editing sites and human for mouse editing sites). To facilitate more detailed searches, we have made the entire database contents available as flat files on the Download web page.

**Figure 2.**
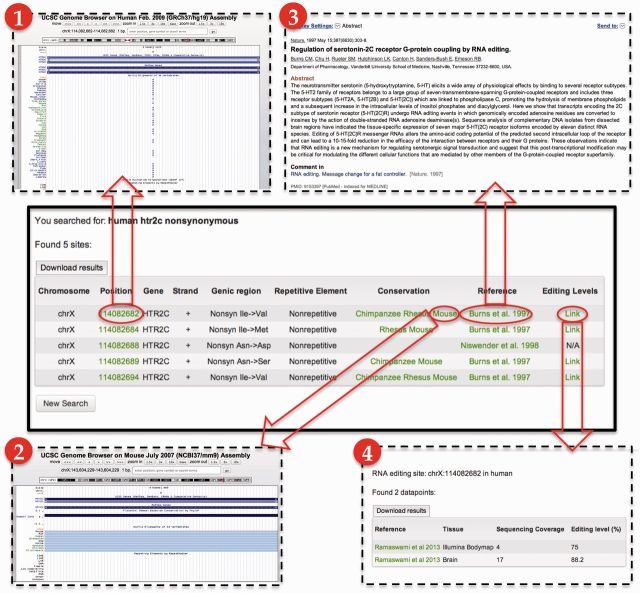
Example of a RADAR search result. A search of human non-synonymous editing sites in the HTR2C gene is displayed. Hyperlinks exist in the following four columns: position, conservation, reference and editing levels. (1) Clicking on the position column will direct the user to the location of the editing site in the UCSC browser. (2) Clicking on a species name in the conservation column will direct the user to the location of the conserved editing site in the UCSC browser. (3) Clicking on the reference column will direct the user to the PubMed abstract for the study that identified the editing site. (4) Clicking on the editing level column will direct the user to tissue-specific editing level measurements for the editing site.

An example results page is displayed in Figure 2. The search parameters are repeated across the top of the page. Information about each editing site is displayed in a single row consisting of nine columns: chromosome, position, gene, strand, genic region, repetitive element, conservation, reference and editing levels. Clicking on the ‘position’ column will direct the user to this location in UCSC genome browser displaying the overlapping gene annotations, genomic nucleotide conservation, overlapping SNP database entries and overlapping repetitive elements. Clicking on an organism under the conservation column will direct the user to the UCSC genome browser location of the conserved editing site in the selected organism. Clicking on the reference column will direct the user to the PubMed abstract for the selected study. Users can download their search results as a tab-delimited text file by clicking on the ‘Download results’ button. A more detailed explanation of the results page can be found on the Tutorial web page.

Tissue-specific editing levels from RNA-seq data (23,25–27,29–31) are available by clicking on the ‘link’ in the ‘editing levels’ column. The information from a single experiment is displayed in each row, which consists of four columns: link to the PubMed abstract for that study, tissue studied, sequencing coverage and editing level. At the time of first release, RADAR contains 1 343 464 human, 7272 mouse and 3155 fly tissue-specific editing level measurements of 975 734 human, 7272 mouse and 2698 fly editing sites, respectively.

### Database architecture and web interface

RADAR was built using the Django web framework coupled with a backend MySQL database. The web page was published using an Apache server hosted by Amazon Web Services. RADAR is freely accessible at http://RNAedit.com.

## DISCUSSION AND FUTURE DIRECTIONS

The recent boom in A-to-I editing site identification has necessitated the development of RNA editing databases to help elucidate the biological functions of specific editing sites. The major advantages of RADAR over DARNED are the comprehensive compilation of A-to-I editing sites, the curation of extensive annotations and the gathering of tissue-specific editing level measurements for each editing site. RADAR contains ∼1.4 million human editing sites, which is a substantial increase over the ∼600 000 editing sites in DARNED. Furthermore, RADAR allows users to search for specific subsets of editing sites using any combination of five annotations: genomic location, gene, genic location, repetitive elements and/or editing conservation, whereas DARNED searches are restricted to sequence context or any combination of three annotations: genomic location, gene and genic location. Finally, the catalog of tissue-specific editing levels will help shed light on which biological contexts each editing site may be involved in. The major advantages of DARNED over RADAR are implementation of sequence-based searches, dbSNP identifiers and links to Wikipedia annotations. We are open to implementing similar features in RADAR if so requested by users.

We anticipate that the continued development of high-throughput sequencing technologies will result in numerous new investigations into A-to-I editing in various physiological and pathological contexts. Recent evidence has already linked dysfunction of A-to-I editing with a myriad of human diseases such as cancer (33) and autoimmune disorders (34). As more data are generated and included, RADAR will provide a centralized repository providing information on the locations and dynamic regulation of A-to-I editing sites in the transcriptome of metazoans.

## FUNDING

Stanford Genome Training Program and Stanford Graduate Fellowship (to G.R.). The U.S. National Institutes of Health [GM102484 to J.B.L.]. Funding for open access charge: National Institutes of Health.

*Conflict of interest statement*. None declared.
